# Kinematic Contribution to Javelin Velocity at Different Run-Up Velocities in Male Athletes

**DOI:** 10.5114/jhk/168143

**Published:** 2023-07-06

**Authors:** Mizuki Makino, Koichi Nakayama, Yuka Ando, Kenji Tauchi

**Affiliations:** 1 Graduate School of Health and Sport Sciences, Chukyo University, Aichi, Japan.; 2 School of Health and Sport Sciences, Chukyo University, Aichi, Japan.

**Keywords:** mechanical energy, javelin throw, forward velocity, upward velocity, three-dimensional motion analysis

## Abstract

In javelin training, many athletes improve their throwing technique by throwing from a slower run-up velocity than in competitions. However, whether the acquisition of javelin velocity in throwing from a slower run-up velocity is the same as in full run-up throwing is unclear. The purpose of this study was to clarify the differences in the contribution of each movement to the javelin velocity caused by changes in the run-up velocity within an individual. Twelve collegiate male javelin throwers were included in this study. Athletes performed two types of throws: one-cross throwing (Cross) and full run-up throwing (Run). The coordinates of reflective markers attached to the thrower’s body and the javelin were recorded using an optical motion capture system. The percentage contribution of each joint movement to the javelin velocity was calculated and compared between Cross and Run. Cross had a lower contribution of trunk forward lean to forward and upward javelin velocities compared to Run. On the other hand, Cross had a higher contribution of trunk counter-clockwise rotation to forward and upward javelin velocities than Run. These results suggest that as the velocity of run-up changes within an individual, the acquisition of javelin velocity also changes.

## Introduction

A javelin throw is a track and field event in which the athlete throws a javelin after a run-up and competes for distance. Many studies have shown that the distance of the javelin throw is strongly related to the velocity of the javelin at the time of release (release velocity) (Komi and Mero, 1985; Mero et al., 1994; Murakami et al., 2006; Whiting et al., 1991). The appropriate release angle for the javelin is about 30° (Hubbard and Alaways, 1987). Therefore, to throw the javelin further, it is necessary to simultaneously increase both the forward and upward release velocities, thereby increasing the resultant release velocity while maintaining a suitable release angle (Makino and Tauchi, 2022). In the javelin throw, it is considered that the thrower first acquires whole-body momentum (mechanical energy) by the run-up, then increases the velocity of the upper torso, by slowing the legs and the lower torso, and accelerates the throwing arm and the javelin with a whip-like movement (Bartonietz, 2000). Furthermore, biomechanical studies on the javelin throw have revealed that javelin throwers with superior records have a faster center of gravity velocity at the last rear foot touchdown (Bartlett et al., 1996; Murakami et al., 2017). Given these considerations, it seems that in order to throw the javelin farther, it is important to increase the whole-body mechanical energy by making the run-up faster, and to increase the release velocity by the mechanism shown by Bartonietz (2000).

However, javelin throwers do not throw from the same run-up velocity as in competitions during their daily training throws. Many javelin throwers work on improving their throwing technique by throwing from a slower run-up velocity than in competitions. Additionally, the mechanical energy of the whole body is transferred to the javelin after the final left-foot touchdown (in right-handed throwers) (Morriss et al., 2001). This suggests that the slower the run-up velocity, the smaller the kinetic energy in the whole body (Bartonietz, 2000), and the smaller the kinetic energy ultimately transferred to the javelin. However, previous studies on the javelin throw have not clarified how the forward and upward release velocities change with a decrease in the run-up velocity.

Furthermore, whether the acquisition of javelin velocity in throws from a slower run-up velocity differs from that in throws from a competitive run-up velocity (full run-up throwing) is unclear. If the acquisition of javelin velocity in throws from a slower run-up velocity differs significantly from that of a full run-up throw, then throwers and coaches need to understand these differences and adjust the run-up velocity of throws in training.

Sprigings et al. (1994) proposed a method to quantify the racket velocity acquired by each movement by the outer product of each angular velocity vector of the body and the displacement vector from its center of rotation to the racket for the tennis serve. In addition, Elliott et al. (1995) evaluated the “contribution” of each movement to racket velocity by relativizing the racket velocity acquired by each body movement with the racket velocity at the same moment. This contribution is a relative assessment of how much velocity was acquired by each movement of the body in the subject part. Therefore, using the method established by Elliott et al. (1995) to quantify the contribution of each movement to javelin velocity when throwing at different run-up velocities, we can clarify how javelin velocity is acquired when throwing from a slow run-up velocity. This clarification will provide useful information for considering how throwing from a slow run-up velocity can be incorporated into training for javelin throws.

Based on the above, the purpose of this study was to clarify the differences in the contribution of each movement to javelin velocity caused by changes in the run-up velocity of a particular athlete. We hypothesized that a slower run-up velocity would be associated with a higher relative contribution from the trunk and the throwing arm to javelin velocity.

## Methods

### Participants

Twelve collegiate male javelin throwers were included in this study (body height: 1.78 ±0.03 m, body mass: 75.6 ± 6.5 kg, age: 19.5 ± 1.5 years, personal best record: 59.3 ± 8.75 m [46.68–71.23 m]). Prior to the experiment, all participants were informed of the purpose of the study and the experimental procedures, and their written consent was obtained. This study was conducted with the approval of the Ethics Committee of the Chukyo University (protocol code 2021-51, approval date: 15 February 2022).

### Design and Procedures

**Figure 1:**
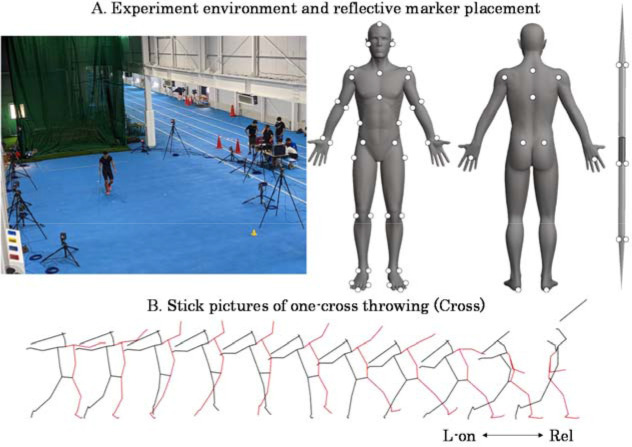
Experimental environment and representative illustration of one-cross throwing. The markers affixed to the body were located at the parietal, ear, superior border of the sternum, C7, xiphoid process, T10, inferior end of the ribs, superior anterior iliac spine, superior posterior iliac spine, hand, lateral and medial side of the wrist, lateral and medial side of the elbow, front and back side of the shoulder, acromion, toe, lateral and medial side of the ball, heel, lateral and medial side of the ankle, lateral and medial side of the knee, and the greater trochanter.

The experiment was conducted in an indoor throwing practice field (Figure 1A). Prior to the experiment, participants were allowed to warm up sufficiently without restriction. The equipment used in the experiment was a men’s javelin (SUPER, NISHI). The experiment was conducted wearing the same spikes as during usual training. Participants performed the following two types of throws, twice each, at maximum effort: a one-cross throw that the athlete throws from a one-step run-up (Cross, Figure 1B), and a full run-up throw that the athlete throws in the same way as in an actual competition (Run). Of the two throws of each type, the throw with the highest resultant velocity of the grip of the javelin at the time of release was used for further analysis.

The coordinates of reflective markers affixed to 47 anatomical landmarks of the body and six javelin points (Figure 1A) during the experimental trials were recorded using a motion capture system (VICON Mx, Vicon Motion System, 14 cameras, 250 Hz). The global coordinate system’s X, Y, and Z axes were defined as the direction to the right of the throwing direction, the throwing direction, and the upward direction, respectively. The 3D coordinates of reflective markers were smoothed using a Butterworth low-pass digital filter with an optimal cutoff frequency (7.5–20 Hz) determined by residual analysis (Winter, 2009). The analysis phase was from the last fore foot touchdown (L-on) to the javelin release (Rel) (Figure 1B).

**Figure 2:**
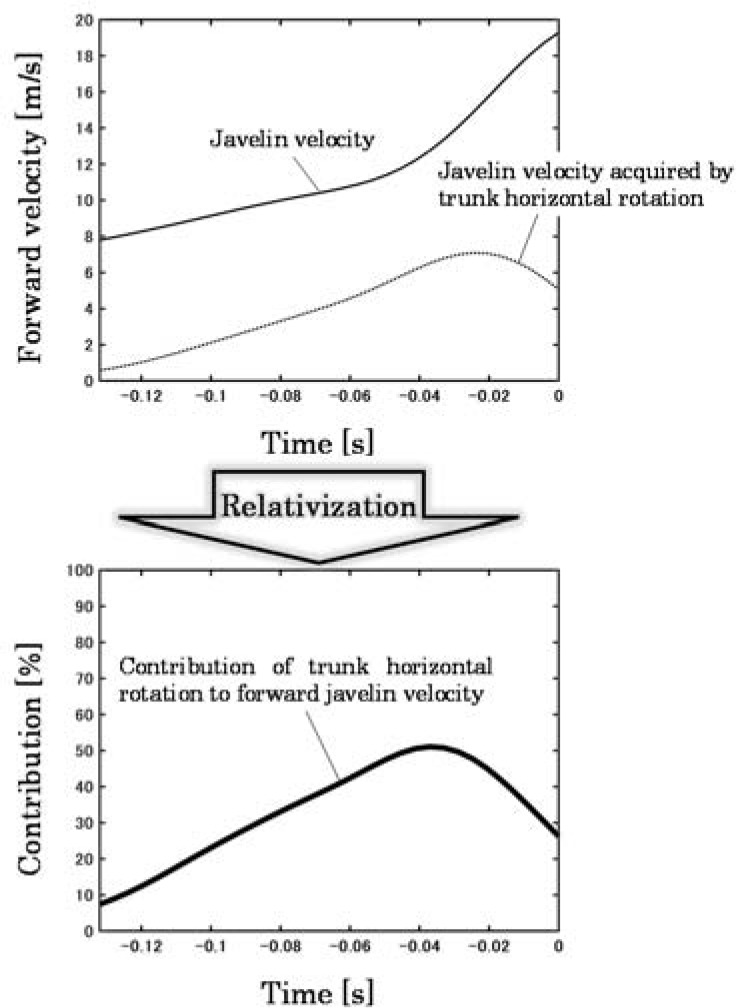
Calculation of the contribution to javelin velocity.


In this study, the whole-body center-of-gravity (CG) velocity at L-on and the whole-body mechanical energy at L-on (*E_whole_*) as well as the mechanical energy at Rel (*E_jav_*) were calculated. Body segment mass and the moment of inertia were calculated using body segment coefficients obtained from youth Japanese athletes (Ae et al., 1992). The mass and moment of inertia around the short axis of the javelin were determined based on a previous study (Maeda et al., 1990) that reported the static characteristics of the same type of the javelin as used in this study (mass: 811 g, moment of inertia around the short axis: 0.41 kg∙m^2^). Rotation around the longitudinal axis of the javelin was assumed to be negligible in this study. The transfer efficiency of the mechanical energy of the javelin was defined as the ratio of the mechanical energy of the javelin at Rel to the whole-body mechanical energy at L-on. The forward and upward release velocities were calculated by differentiating the midpoints of two reflective markers affixed to the grip of the javelin with time, and the values at the time of release were adopted. The release angle was calculated from the forward and upward release velocities. In addition, the method of Elliott et al. (1995) was used as a reference to calculate the javelin velocity acquired by each joint movement (forward-backward lean, leftward-rightward lean, and horizontal rotation of the trunk; adduction-abduction, horizontal adduction-abduction, internal-external rotation of the shoulder; and flexion-extension of the elbow) and the percentage (contribution) of the javelin velocity acquired by each joint movement to the javelin velocity at the same moment was calculated. For example, the contribution of the trunk joint coordinate system’s movement around the ***k***-axis (horizontal rotation) to the javelin velocity can be calculated from the following equations (Figure 2):

$$v_{ut\_k}=\omega_{ut\_k}\times r_{j/ut}$$

$$Contibution_{ut\_k}=\frac{v_{ut\_k}}{v_{jav}}.100$$

where $\nu_{ut\_k},\omega_{ut\_k},r_{j/ut},\text{ and }v_{jav}$ are the javelin velocity acquired by trunk horizontal rotation, angular velocity vector of trunk horizontal rotation in the global coordinate system, displacement vector from the trunk joint (center of the lower rib end) to the grip of the javelin, and javelin velocity, respectively. The segment (upper torso, upper arm, and forearm) and joint (trunk, shoulder, and elbow) coordinate systems were defined based on the studies by Murata et al. (2022) and Kariyama et al. (2016). The contribution of the lower limb and lower torso movements was evaluated by relativizing the velocity of the midpoint of the lower end of both ribs by the javelin velocity. Since the release velocity in the forward and upward directions is strongly related to the throwing record in the javelin (Makino and Tauchi, 2022), the lateral direction was excluded. The joint motions of pronation/supination of the elbow joint and palmar/dorsal and radial/ulnar flexion of the wrist joint were not calculated because the radius relative to the joint angular velocity vector was small.

### Statistical Analysis

The normality of each variable was confirmed using the Shapiro-Wilk test. Differences in variables between Cross and Run were confirmed using a paired *t*-test for those that followed a normal distribution and the Wilcoxon signed rank test for those that did not (α = 0.05). Hedges’ g was calculated as the effect size (ES) from the following equation:


$$Hedges'\text{g}=\left(\frac{\bar{X_{C}}-\bar{X_{R}}}{SD_{p}}\right)\times \left(1-\frac{3}{4\left(n_{C}+n_{R}\right)-9}\right)$$

where $\bar{X_{C}}$,$\bar{X_{R}}$, *SD_p_, n_c_, n_R_* are the mean in Cross and Run, pooled standard deviation, and the number of samples in Cross and Run, respectively. The threshold for determining the magnitude of ES were 0.20, 0.50, and 0.80 for small, moderate, and large, respectively, with absolute value of ES < 0.20 considered trivial (Lakens, 2013). Differences in contributions to javelin velocity were confirmed using a paired *t*-test in statistical parametric mapping (α = 0.05).

## Results

Table 1 shows the mean ± standard deviation (SD) of various variables for Cross and Run. In Cross, the CG velocity at L-on, the whole body’s mechanical energy at L-on, and the javelin at Rel showed significantly lower values than in Run. On the other hand, the transfer efficiency was significantly higher in Cross than in Run. In addition, Cross showed significantly lower forward release velocity and higher values for the release angle than Run.

**Table 1:** Mean ± standard deviation of variables in Cross and Run.

Variables		Cross			Run			*p*	*Hedges’ g*	effect
Selected variables										
CG velocity at L-on	[m/s]	3.3	±	0.2	5.3	±	0.5	<0.001^*^	−5.38	Large
*E_whole_* at L-on	[J]	1140.6	±	59.2	1832.4	±	199.1	0.002^*, a^	−4.67	Large
*E_jav_* at Rel	[J]	159.2	±	21.2	205.0	±	28.5	<0.001^*^	−1.81	Large
Efficiency	[%]	14.0	±	1.7	11.2	±	1.4	<0.001^*^	1.75	Large
Release variables										
Forward velocity	[m/s]	15.7	±	1.5	18.7	±	1.6	<0.001^*^	−1.87	Large
Upward velocity	[m/s]	10.1	±	1.3	10.5	±	1.0	0.115	−0.40	Small
Release angle	[deg]	32.7	±	4.5	29.4	±	2.9	0.009^*^	0.85	Large

* : Significant difference (p < 0.05) *a: Wilcoxon signed-rank test*

**Figure 3:**
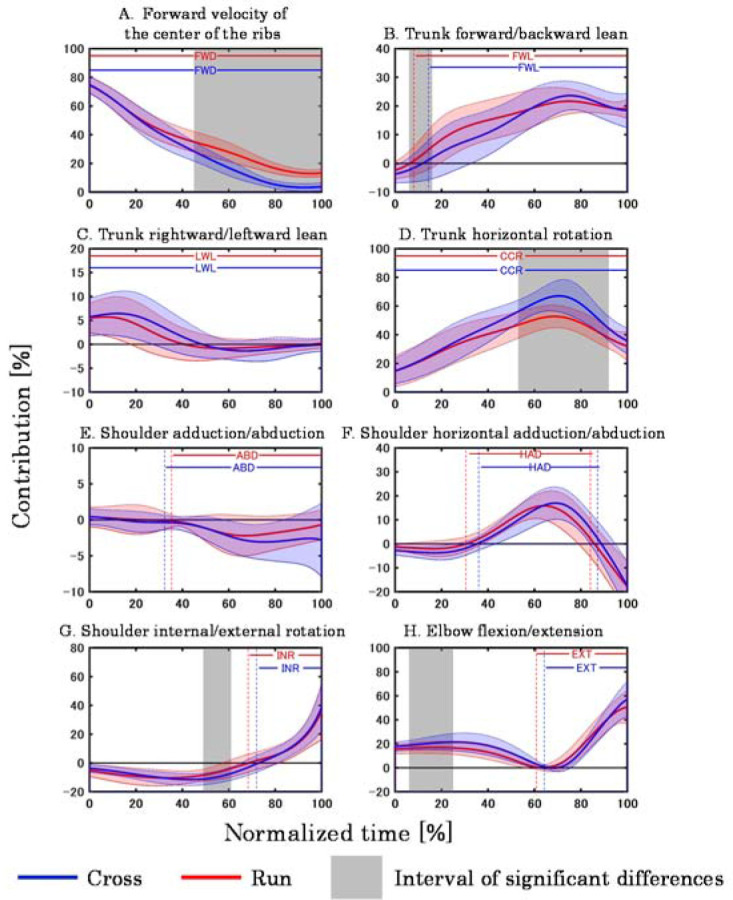
Contribution of each movement to the forward javelin velocity. The alphabets in the figures indicate the mean interval for each throw in which the movement was mainly performed to accelerate the javelin (FWD: forward movement of the center of the ribs, FWL: trunk forward lean, LWL: trunk leftward lean, CCR: trunk counter-clockwise rotation, ABD: shoulder abduction, HAD: shoulder horizontal adduction, INR: shoulder internal rotation, EXT: elbow extension).

Figure 3 shows the mean ± SD in the contribution to the forward javelin velocity. Compared to Run, Cross had significantly lower contributions to forward javelin velocity due to the forward velocity of the center of the lower limbs (45–100%), forward lean of the trunk (6–16%), and external rotation of the shoulder (49–61%). In contrast, Cross had significantly higher contributions to forward javelin velocity due to counter-clockwise rotation of the trunk (53–92%) and flexion of the elbow (6–25%) than Run.

**Figure 4:**
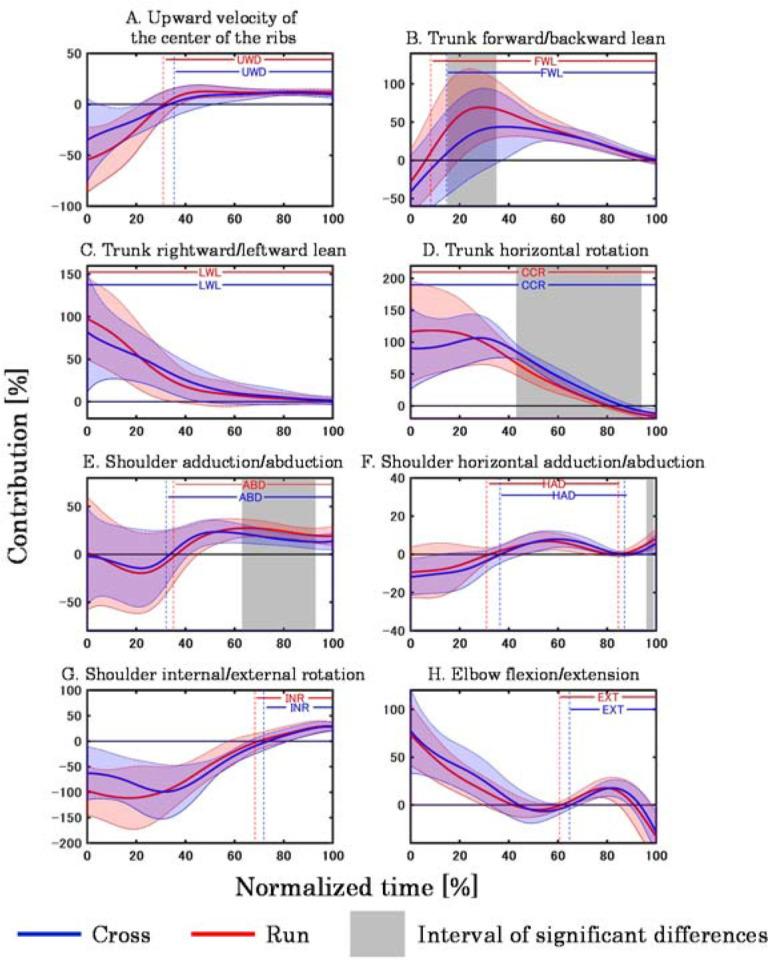
Contribution of each movement to the upward javelin velocity. The alphabets in the figures indicate the mean interval for each throw in which the movement was mainly performed to accelerate the javelin (UWD: upward movement of the center of the ribs, FWL: trunk forward lean, LWL: trunk leftward lean, CCR: trunk counter-clockwise rotation, ABD: shoulder abduction, HAD: shoulder horizontal adduction, INR: shoulder internal rotation, EXT: elbow extension).

Figure 4 shows the mean ± SD in the contribution to the upward javelin velocity. Cross had significantly lower contributions to upward javelin velocity due to forward lean of the trunk (15–35%), abduction (63–93%), and horizontal abduction of the shoulder (96–99%) compared to Run. In contrast, Cross had a significantly higher contribution to upward javelin velocity due to counter-clockwise rotation of the trunk (43–94%) than Run.

## Discussion

In the present study, we found that Cross had a lower CG velocity at L-on than Run. Cross showed a lower contribution of the forward velocity of the center of ribs and the forward lean of the trunk and a higher contribution of the counter-clockwise rotation of the trunk to forward javelin velocity than Run. In addition, Cross showed a lower contribution of the forward lean of the trunk and abduction of the shoulder and a higher contribution of the counter-clockwise rotation of the trunk to upward javelin velocity than Run. These results differed from the hypothesis of the present study that a slower run-up velocity would be associated with a higher relative contribution from the trunk and the throwing arm to javelin velocity.

First, the whole-body’s mechanical energy at L-on was smaller in Cross than in Run (Table 1). Kinetic energy, a factor of mechanical energy, is determined by half the product of mass and velocity squared. In other words, in Cross, where the whole-body’s CG velocity was lower than in Run (Table 1), the whole-body’s mechanical energy was correspondingly reduced.

Furthermore, the javelin’s mechanical energy at Rel was smaller in Cross than in Run (Table 1). The whole body’s mechanical energy is believed to be transferred to the javelin after the L-on (Morriss et al., 2001). Based on this, it can be inferred that because Cross had small whole-body’s mechanical energy at L-on compared to Run, mechanical energy ultimately transferred to the javelin was likewise small. However, the efficiency of energy transfer was higher in Cross than in Run (Table 1). In other words, in the javelin throw, the ratio of energy transferred from the body to the javelin decreased as the run-up speed increased.

Next, focusing on the release variables, Cross had a lower forward release velocity than Run, and no significant difference was observed in the upward release velocity (Table 1). This suggests that both forward and upward release velocities do not increase or decrease linearly with changes in intra-individual run-up velocity in the javelin throw. In addition, the release angle is determined by the forward and upward release velocity ratios. Therefore, the higher release angle observed in Cross than in Run (Table 1) may have been caused primarily by the decreased forward release velocity in Cross.

Focusing on the contribution to the forward javelin velocity, Cross had a lower contribution of forward velocity of the center of ribs in the second half of the phase than Run (Figure 3A). The forward velocity of the center of ribs in the javelin throw is a factor that is increased by the whole body moving forward by the run-up. Thus, we believe that the forward velocity of the center of the ribs was not higher in Cross than in Run, because there was only one step to the run-up in Cross, and the contribution to the forward javelin velocity was correspondingly lower. Furthermore, at Rel, the contributions of forward velocity of the center of ribs in Cross and Run were 3.7 ± 2.8 % and 13.4 ± 2.7 %, respectively. This clarifies that in Cross, most of the forward release velocity was acquired by the trunk and throwing arm movements, whereas in Run, about one-eighth of the forward release velocity was acquired by the forward velocity of the center of the ribs.

The contribution of the forward lean of the trunk to the forward and upward javelin velocities was lower in Cross than in Run (Figures 3B, 4B). The forward lean of the trunk in the javelin throw is thought to be caused by a decrease in left knee flexion at L-on which effectively converts the momentum of the whole body into trunk rotation (Bartlett and Best, 1988; Bartonietz, 2000; Morriss and Bartlett, 1996). However, the perspectives of these previous studies differ from those of the present study in that they only focused on full run-up throwing. Based on the difference in CG velocity, the momentum of the whole body at L-on in Cross was smaller than in Run. Therefore, even if the left knee flexion at L-on was reduced in Cross, the trunk leant forward only slightly because the amount of momentum converted into the trunk rotation was small. Given this, it is possible that less whole-body momentum was observed at L-on in Cross and that the forward lean of the trunk was subsequently less than in Run, with a concomitant decrease in their contribution to the forward and upward javelin velocities. In addition, Morriss et al. (2001) reported that the total amount of whole-body’s mechanical energy was clearly reduced after L-on. This means that not only is mechanical energy transferred from the lower limbs to the trunk after L-on, but also more energy is absorbed. In other words, the lower energy transfer efficiency of Run compared to Cross (Table 1) may be due to the greater energy absorbed by the body after L-on.

In addition, Cross had a lower contribution of shoulder abduction to the upward javelin velocity than Run (Figure 4E). Shoulder abduction is the most effective movement in the shoulder joint for moving the javelin upward. In the javelin, athletes acquire javelin velocity by transferring the whole-body mechanical energy obtained in the run-up from the torso to the throwing arm (Campos et al., 2004; Mero et al., 1994; Whiting et al., 1991). Therefore, it is likely that in Cross, where the whole-body mechanical energy at L-on was less than in Run (Table 1), the energy transferred from the trunk to the throwing arm was also less, thus decreasing the contribution of shoulder abduction to the upward javelin velocity.

**Figure 5:**
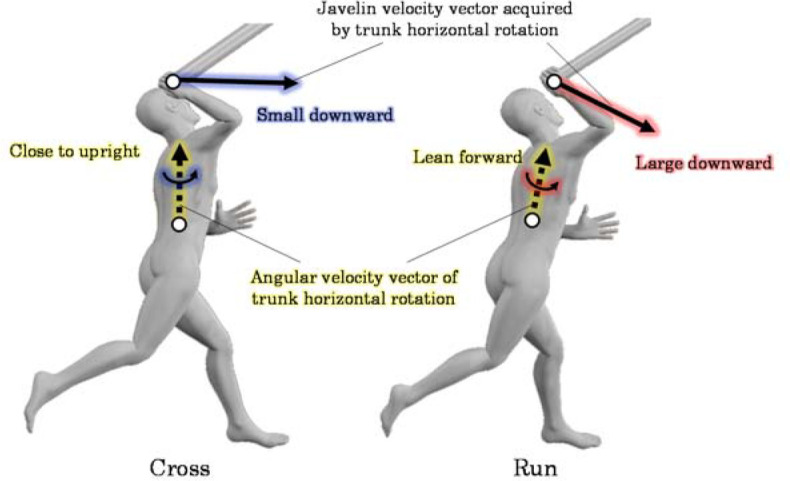
A typical example of throwing movement in Cross and Run before Rel (90% normalized time) in one participant.

In contrast to previous trends, the contribution of the counter-clockwise rotation of the trunk to the forward and upward javelin velocities was higher in Cross than in Run (Figures 3D, 4D). This may be related to the fact that in Cross, the forward-leaning of the trunk after L-on was not more than that in Run. A larger angle of the trunk forward lean after L-on is associated with an increased forward release velocity (Makino and Tauchi, 2022). Therefore, it is possible that in Run, where the forward release velocity was higher than in Cross, the angle of the trunk forward lean after L-on was large. A significant difference was confirmed in the posture of one participant at 90% normalized time, with the trunk being close to upright in Cross while leaning forward in Run (Figure 5). The larger angle of the trunk forward lean means that the angular velocity vector of the trunk counter-clockwise rotation was more oriented in the throwing direction. In such a posture, the counter-clockwise rotation causes the javelin to move obliquely downward. On the other hand, when the trunk is upright, its counter-clockwise rotation is a factor in moving the javelin forward. Therefore, the contribution of the counter-clockwise rotation of the trunk to the forward and upward javelin velocities was considered higher in Cross than in Run because the trunk was more upright and rotated counter-clockwise.

Finally, there are some limitations in the present study. First, this study does not clarify the specific differences in movement between Cross and Run. Future research should examine the actual changes in movement (angle and angular velocity) that may have caused the differences in contribution. Second, since the present study shows the percentage of contribution to the javelin velocity, it is not clear which movements were responsible for the change in release velocity. To clarify this, it is necessary to directly examine the javelin velocity acquired by each movement. Third, the present study describes the transfer of mechanical energy, but the details are not clear. Future research should analyze energetics such as joint power.

## Conclusions

Cross had a lower CG velocity of the whole body at L-on than Run. Cross had a lower contribution of the forward velocity of the center of ribs to forward javelin velocity, shoulder abduction to upward javelin velocity, and trunk forward lean to forward and upward javelin velocities than Run. On the other hand, Cross had a higher contribution of the trunk counter-clockwise rotation to the forward and upward javelin velocities than Run. These results indicate that Cross tends to emphasize the contribution of rotational motion to javelin velocity, whereas Run tends to emphasize more linear movements.

## References

[ref1] Ae, M., Tang, H. & Yokoi, T. (1992). Estimation of inertia properties of the body segments in Japanese athletes. Society of Biomechanism Japan, 11, 23–33. 10.3951/biomechanisms.11.23

[ref2] Bartlett, R. & Best, R. (1988). The biomechanics of javelin throwing: A review. Journal of Sports Sciences, 6(1), 1–38. 10.1080/02640418808729791 3043013

[ref3] Bartlett, R., Müller, E., Lindinger, S., Brunner, F. & Morriss, C. (1996). Three-dimensional evaluation of the kinematic release parameters for javelin throwers of different skill levels. Journal of Applied Biomechanics, 12(1), 58–71. 10.1123/jab.12.1.58

[ref4] Bartonietz, K. (2000). Javelin throwing: an approach to performance development. In V.M. Zatsiorsky (Ed.), Biomechanics in sport: performance enhancement and injury prevention, Blackwell Science, 401–434.

[ref5] Campos, J., Brizuela, G. & Ramón, V. (2004). Three-dimensional kinematic analysis of elite javelin throwers at the 1999 IAAF World Championships in Athletics. New Studies in Athletics, 19, 47–57.

[ref6] Elliott, B.C., Marshall, R.N. & Noffal, G.J. (1995). Contributions of upper limb segment rotations during the power serve in tennis. Journal of Applied Biomechanics, 11(4), 433–442. 10.1123/jab.11.4.433

[ref7] Hubbard, M. & Alaways, L.W. (1987). Optimum release conditions for the new rules javelin. Journal of Applied Biomechanics, 3(3), 207–221.

[ref8] Kariyama, Y., Hobara, H. & Zushi, K. (2017). Differences in take-off leg kinetics between horizontal and vertical single-leg rebound jumps. Sports Biomechanics, 16(2), 187–200. 10.1080/14763141.2016.1216160 27593193

[ref9] Komi, P.V. & Mero, A. (1985). Biomechanical analysis of Olympic javelin throwers. Journal of Applied Biomechanics, 1(2), 139–150. 10.1123/ijsb.1.2.139

[ref10] Lakens, D. (2013). Calculating and reporting effect sizes to facilitate cumulative science: a practical primer for t-tests and ANOVAs. Frontiers in Psychology, 4, 863. 10.3389/fpsyg.2013.00863 PMC3840331 24324449

[ref11] Maeda, M., Nomura, H. & Miyagaki, M. (1990). Characteristics of javelin-form and moment of inertia-. Research Quarterly for Athletics, 2, 18–28.

[ref12] Makino, M. & Tauchi, K. (2022). Kinematic factors related to forward and vertical release velocity in male javelin throwers. International Journal of Sport and Health Science, 20, 249–259. 10.5432/ijshs.202146

[ref13] Mero, A., Komi, P.V., Korjus, T., Navarro, E. & Gregor, R.J. (1994). Body segment contributions to javelin throwing during final thrust phases. Journal of Applied Biomechanics, 10(2), 166–177. 10.1123/jab.10.2.166

[ref14] Morriss, C. & Bartlett, R. (1996). Biomechanical factors critical for performance in the Men’s javelin throw. Sports Medicine, 21(6), 438–446. 10.2165/00007256-199621060-00005 8784963

[ref15] Morriss, C., Bartlett, R. & Navarro, E. (2001). The function of blocking in elite javelin throws: A re-evaluation. Journal of Human Movement Studies, 41(3), 175–190.

[ref16] Murakami, M., Tanabe, S., Ishikawa, M., Isolehto, J., Komi, P.V. & Ito, A. (2006). Biomechanical analysis of the javelin at the 2005 IAAF World Championships in Athletics. New Studies in Athletics, 21, 67–80.

[ref17] Murakami, M., Tanabe, S., Ishikawa, M. & Ito, A. (2017). The relationship between approach run kinematics and javelin throwing performance. Asian Journal of Coaching Science, 1, 1–14. 10.29426/ajcs.201712_1(1).0001

[ref18] Murata, M., Fujii, N. & Suzuki, Y. (2022). Mechanical energy flow of the racket holding arm in the tennis serve focusing on the energy form. International Journal of Sport and Health Science, 20, 48–65. 10.5432/ijshs.202135

[ref19] Sprigings, E., Marshall, R., Elliott, B. & Jennings, L. (1994). A three-dimensional kinematic method for determining the effectiveness of arm segment rotations in producing racquet-head speed. Journal of Biomechanics, 27(3), 245–254. 10.1016/0021-9290(94)90001-9 8051185

[ref20] Whiting, W.C., Gregor, R.J. & Halushka, M. (1991). Body segment and release parameter contributions to New-rules javelin throwing. International Journal of Sport Biomechanics, 7(2), 111–124.

[ref21] Winter, D.A. (2009). *Biomechanics and motor control of human movement* (4th ed.). New Jersey: John Wiley & Sons.

